# Thalamic local field potentials recorded using the deep brain stimulation pulse generator

**DOI:** 10.1016/j.cnp.2022.03.002

**Published:** 2022-03-10

**Authors:** A.W.G Buijink, D.A. Piña-Fuentes, M.J. Stam, M. Bot, P.R. Schuurman, P. van den Munckhof, A.F. van Rootselaar, R.M.A. de Bie, M. Beudel

**Affiliations:** a Department of Neurology, Amsterdam University Medical Centers, Amsterdam Neuroscience, University of Amsterdam, Amsterdam, The Netherlands; b Department of Neurosurgery, Amsterdam University Medical Centers, Amsterdam Neuroscience, University of Amsterdam, Amsterdam, The Netherlands

**Keywords:** Tremor, Adaptive, Closed-loop, Deep brain stimulation, Essential tremor

## Abstract

**Background:**

Essential tremor (ET) is one of the most common movement disorders, and continuous deep brain stimulation (DBS) is an established treatment for medication-refractory cases. However, the need for increasing stimulation intensities, with unpleasant side effects, and DBS tolerance over time can be problematic. The advent of novel DBS devices now provides the opportunity to longitudinally record LFPs using the implanted pulse generator, which opens up possibilities to implement adaptive DBS algorithms in a real-life setting.

**Methods:**

Here we report a case of thalamic LFP activity recorded using a commercially available sensing-enabled DBS pulse generator (Medtronic Percept PC).

**Results:**

In the OFF-stimulation condition, a peak tremor frequency of 3.8 Hz was identified during tremor evoking movements as assessed by video and accelerometers. Activity at the same and supraharmonic frequency was seen in the frequency spectrum of the LFP data from the left vim nucleus during motor tasks. Coherence analysis showed that peripherally recorded tremor was coherent with the LFP signal at the tremor frequency and supraharmonic frequency.

**Conclusion:**

This is the first report of recorded tremor-related thalamic activity using the electrodes and pulse generator of an implanted DBS system. Larger studies are needed to evaluate the clinical potential of these fully implantable systems, and ultimately pulse generators with sensing-coupled algorithms driving stimulation, to really close the loop.

## Highlights

• Novel DBS pulse generators can longitudinally record local field potentials.
• We report tremor-related thalamic activity recorded using an implanted DBS system.
• This opens up possibilities to implement adaptive DBS into clinical practice.

## 1 Introduction

Essential tremor (ET) is one of the most common movement disorders, and continuous deep brain stimulation (cDBS) is an effective treatment for medication-refractory essential tremor (Ferreira et al., 2019). However, the need for increasing stimulation intensities, with unpleasant side effects, and tolerance to cDBS over time can be problematic (Fasano and Helmich, 2019). Recently, adaptive deep brain stimulation (aDBS) algorithm for suppression of essential tremor have been proposed (He et al., 2021, Opri et al., 2020). In these studies, tremor-provoking movement states were successfully detected using either electrocorticography or local field potentials (LFPs) recorded from the same electrodes implanted for stimulation. Using these algorithms, suppression of tremor was achieved while delivering less than 40% of the energy used for conventional DBS. These approaches are very effective but either used externalized electrodes (He et al., 2021) or different electrodes for sensing and stimulation (Opri et al., 2020). The advent of novel DBS devices now provides the opportunity to longitudinally record LFPs using the implanted pulse generator, which opens up possibilities to implement these algorithms in a real-life setting. Here we report a case of thalamic LFP activity recorded using a commercially available sensing-enabled DBS pulse generator (Medtronic Percept PC). Our goal is to assess the feasibility of extracting usable neurophysiological biomarkers, recorded from the thalamic ventrointermediate (Vim) nucleus (Cagnan et al., 2019).

## 2 Methods

### 2.1 Patient characteristics

We describe a 70-years old male patient, diagnosed with essential tremor based on the Consensus Statement on the Classification of Tremors (Bhatia et al., 2018) at the age of 30. The patient underwent DBS surgery 6 years previously. Electrodes (lead model 3389, Medtronic, Minneapolis, MN) were placed in a trajectory encompassing both the thalamic Vim nucleus and the posterior subthalamic area (PSA). The left dorsal electrode contact position for ACPC-aligned MRI relative to the midcommissural point was x = 13.3, y = −4.7, z = 2.0 in mm. Ventral electrode contact position was x = 9.9, y = −6.9, z = −3.1. For the right side the dorsal electrode contact position was x = 14.4, y = −5.3, z = 1.0. Ventral position was x = 10.9, y = −7.1, z = −4.2. The following years the patient had good tremor suppression. On the left side monopolar cathodal stimulation (ventromedial contact (L1), voltage 1.7 V, pulse width 60us, stimulation frequency 130 Hz) was active and well-tolerated. On the rightside bipolar stimulation (ventromedial contact (cathodal, R9) and dorsomedial contact (anodal, R10) montage with voltage 3.3 V, pulse width 60us, and stimulation frequency 130 Hz) was active. After battery depletion, the pulse generator was replaced with a Percept PC neurostimulator (Medtronic, Minneapolis, USA) in the context of standard clinical care due, and place over the right pectoral muscle. The patient was asked to visit our clinic 4 weeks after the battery replacement for the assessment of LFPs.

### 2.2 Data acquisition

LFPs were recorded in a bipolar mode from both electrodes with a sampling frequency of 250 Hz. For pragmatic reasons, the contacts surrounding the contact used as a cathode for stimulation were chosen for this recording, since the ON stimulation condition would in this case reflect the patients best clinical response to DBS. For the left electrode using contact pair ventral and dorsomedial (0–2), for the right electrode using the similar contact pair (8–10). This resulted in two bipolar channels – one channel per hemisphere. The raw LFPs were amplified by 250×, and continuously streamed via a communicator (model 8880T2) to the clinician programmer tablet (model CT900D). Two 3D accelerometers (+/− 3 g, TMS International, Oldenzaal, The Netherlands) were attached to the dorsal surface of both hands in order to measure movements of the hand. Bipolar ECG signal was measured between two electrodes placed on the right and left shoulder. These signals were recorded using a TMSi Porti amplifier (monopolar, common average reference, anti-aliasing low-pass filtering with a cut-off frequency of 500 Hz and sampling frequency of 2048 Hz, TMSi, The Netherlands). All external data running through the TMSi amplifier were imported into Matlab. A USB Webcam was added to the software application toolbox TMSi Polybench to record the experiment on video.

### 2.3 Experimental protocol

During the recording, the patient performed an experimental protocol with pre-specified tasks that included the following items: rest, stretching of the arm, fist opening and closing and finger-to-nose maneuvers with the right and left arm separately, and speech. These tasks were partly based on the Essential Tremor Rating Assessment Scale (TETRAS) items (Elble et al., 2012). The same protocol was executed with stimulation OFF and ON. Tremor severity was assessed using the TETRAS (Elble et al., 2012). This study was approved by the local ethics committee and informed written consent was received from the patient.

### 2.4 Data analysis

Offline analyses were performed in MATLAB (The Mathworks, Natick, MA) using the FieldTrip toolbox (Oostenveld et al., 2011). LFP data was recorded in JSON format, and converted to .mat files using the open-source Perceive Toolbox (https://www.github.com/neuromodulation/perceive/). A recent study showed that LFP data obtained using the Percept PC is often impacted by ECG artefacts (Neumann et al., 2021). In our case, severe ECG artefacts were present in the bipolar recording from the right hemisphere. No ECG artefacts were visually identifiable in the bipolar recording from the left hemisphere. Since the LFP and accelerometer signals are recorded using different systems, data was synchronized with the cross-correlation between the ECG artefact captured in the LFP recordings and the ECG signal. A prerequisite for applying cross-correlation is that both signals have the same sampling frequency. Therefore, the ECG recording was first down-sampled to the sampling frequency of the pulse generator (250 Hz). After applying the cross-correlation, one signal is shifted relative to the other such that the peak of similarity will be at a lag of zero and the signals are synchronized. Subsequently, ECG artefact correction was performed on the LFPs using a custom-made template subtraction method similar to a method developed for ECG-artefact removal from electromyography signals (Costa Junior et al., 2019). In short, epochs were created around the R-peaks, identified using the MATLAB *findpeaks* function, and averaged to create a recording-specific ECG artefact template. On each QRS complex epoch in the original LFP signal, the QRS complex template is optimized by adjusting the parameters, scale and offset, such that the sum of squared error is minimized, using the build-in MATLAB function *lsqnonlin.* This QRS-specific optimized template is then subtracted from the QRS complex epoch in the original LFP signal. The recorded bipolar LFPs and accelerometer signal was band-pass filtered at 1–40 Hz using a forward–backward 4th-order Butterworth bandpass filter. For subsequent analyses the dominant axis of the tremor (Z-axis of the accelerometer) signal was used. Spectrograms were built using a 500 ms window length with 25% overlap. Signals were segmented into 500 ms overlapping 2 s epochs. For each epoch, the Fourier transform was computed using a Hanning window; these were subsequently averaged, and coherence was calculated between tremor signals and LFPs (frequency resolution 0.5 Hz) with the use of the NeuroSpec toolbox (Halliday et al., 1995) (https://www.neurospec.org/). Absolute power amplitudes are reported. For visualization purposes, electrode locations were mapped in MNI space using the Lead DBS toolbox according to standard procedures (Horn et al., 2019). The DISTAL Minimal Atlas and DBS Tractography Atlas were used for 3D visualization (Ewert et al., 2018, Middlebrooks et al., 2020).

## 3 Results

**Fig. 1:**
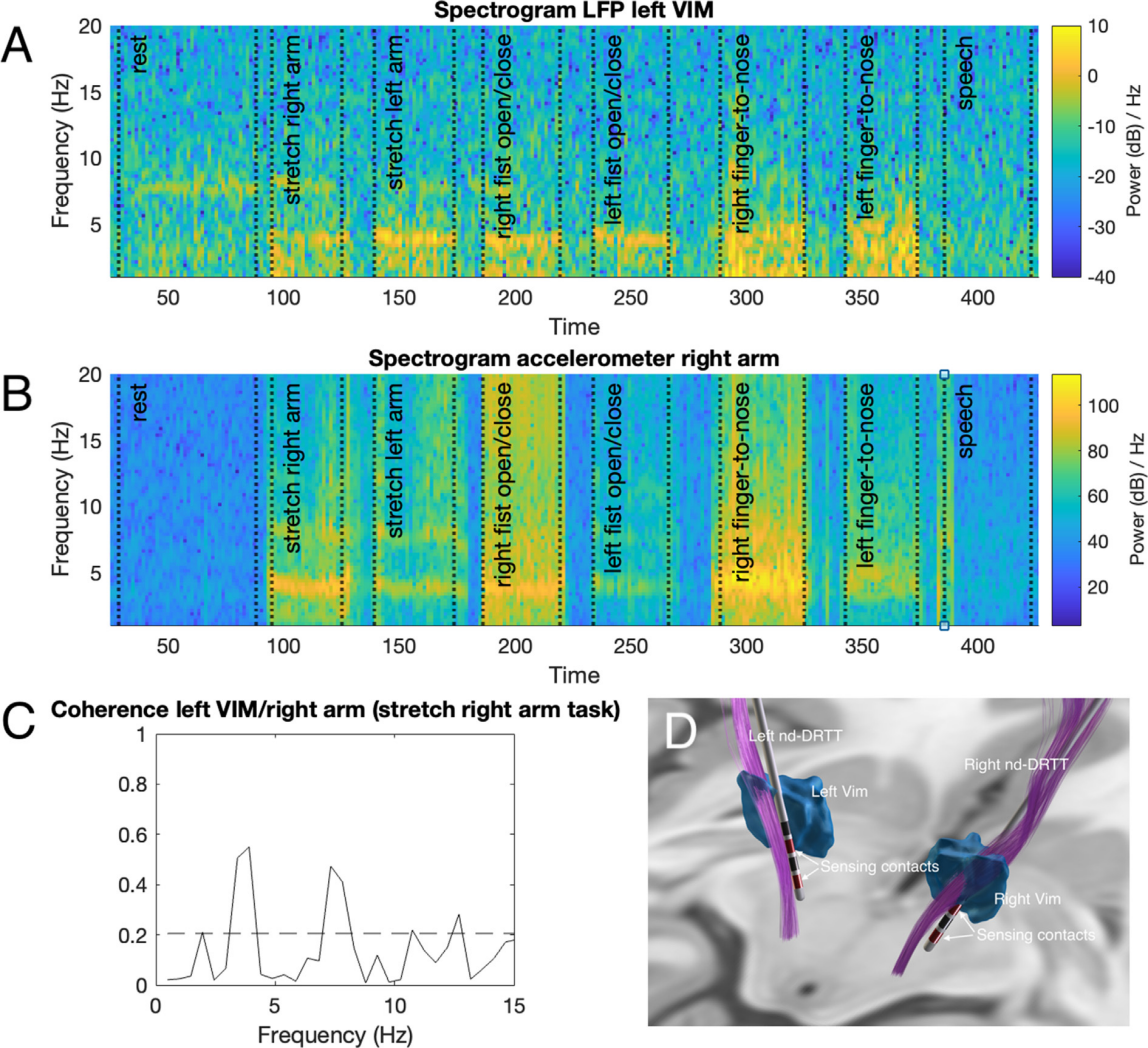
A. Spectrogram of the LFP of the left Vim nucleus with stimulation at 0.0 mA (OFF), tremor activity around 4 Hz is visible throughout the experimental protocol. B. Spectrogram of the dominant accelerometer axis. C. Cross spectral analysis (coherence) of the left Vim and right arm during stretching of the right arm showing significant cortico-kinematic coherence around the tremor frequency and its harmonics. D. Three-dimensional reconstruction of the DBS leads localized in MNI space using Lead-DBS software (Horn et al., 2019). Recording electrode contacts are highlighted in red. *Vim = ventral intermediate nucleus (based on the DISTAL atlas* (Ewert et al., 2018)*), nd-DRTT = non-decussating dentatorubrothalamic tract (based on the DBS Tractography Atlas* (Middlebrooks et al., 2020)*.* (For interpretation of the references to colour in this figure legend, the reader is referred to the web version of this article.)

In the OFF-stimulation condition, a peak tremor frequency of 3.8 Hz was identified during tremor evoking movements as assessed by video and accelerometers (Fig. 1*B*). Activity at the same and supraharmonic frequency was seen in the frequency spectrum of the LFP data from the left vim nucleus during motor tasks (Fig. 1*A and* Supplementary Fig. 1*A*). Coherence analysis showed that peripherally recorded tremor was coherent with the LFP signal at the tremor frequency and supraharmonic frequency (Fig. 1*C)*. In the LFP data from the right vim nucleus, no activity in the tremor frequency range was observed (Supplementary Fig. 1*B*). In the OFF-stimulation condition TETRAS scores were 0/0 during rest, 3.5/4.0 during hand posture and 4/4 during finger-to-nose testing for the right and left side respectively. In the ON-stimulation condition TETRAS scores were 0/0 during rest, 1/1 during hand posture and 2/2.5 during finger-to-nose testing for the right and left side respectively. With a clinically effective stimulation amplitude (1.4 mA for the left vim, 1.2 mA for the right vim) no activity in the tremor frequency range was observed in the LFPs of both the left and right vim (Supplementary Fig. 1*C and 1D*). Data of the entire recording in the OFF and ON condition is presented.

## 4 Discussion

This is the first report of recorded tremor-related thalamic activity using the electrodes and pulse generator of an implanted DBS system. This technological advancement enables measurements of LFPs, and is expected to advance the development of aDBS, such as the algorithm proposed by He and colleagues (He et al., 2021). Previous studies have successfully recorded tremor-related thalamic activity using externalized electrodes, and observed similar coherence between LFPs recorded from the vim nucleus and the contralateral arm using electromyography (He et al., 2021, Marsden et al., 2000, Pedrosa et al., 2014). There are some limitations that need to be addressed. For this case report we have chosen to use the bipolar contact combination surrounding the contact used for stimulation for the recording of LFPs. Future studies with more subjects should assess multiple contact combination, in order to establish whether the presence or absence of tremor activity in LFPs might be related to proximity to the VIM or the dentato-rubro-thalamic tract. Related to this is the fact we have only observed tremor-related activity in the left vim nucleus. This might be related to the proximity of the recording contacts to the VIM or dentato-rubro-thalamic tract. The signal recorded from the right vim was also severely contaminated with ECG-artefacts. Subtraction of ECG artefacts might have a tremendous influence on especially low-frequency activity (Neumann et al., 2021). Based on this N = 1 study, it is difficult to draw any major conclusions regarding the presence or absence of tremor-related vim activity. Finally, we have used ECG-artefacts in the LFP recording of the right vim nucleus to synchronize LFP and accelerometer signals. This method has not been used previously, and might lead to an imprecise synchronization. This might have impacted the coherence analyses. An alternative, more reliable method, might be to use a short period of stimulation ramping at the start of the recording, and synchronize LFP data using the stimulation artefacts in the ECG signals.

This first report of recorded tremor-related thalamic activity using a fully-implantable system for both sensing and stimulation underlines the possibilities of this technology to rapidly advance the development of aDBS. Larger studies are needed to evaluate the clinical potential of these fully implantable systems, and ultimately pulse generators with sensing-coupled algorithms driving stimulation, to really close the loop.

## 5 Author Roles

AWB 1ABC, 2AB, 3A, DPF 1BC, 2AC, 3B, MS 1C, 2B, 3B, PRS 1B, 2C, 3B, MB 1B, 2C, 3B, PM 1B, 2C, 3B, AFR 1B, 2C, 3B, RdB 1B, 2C, 3B, MB 1ABC, 2AC, 3B.

1) Research project: A. Conception, B. Organization, C. Execution;

2) Statistical Analysis: A. Design, B. Execution, C. Review and Critique;

3) Manuscript: A. Writing of the first draft, B. Review and Critique.

## Funding sources

None.

## Disclosures

Prof. de Bie received research grants from Neuroderm, Medtronic, Parkinson Vereniging.

AMC Foundation, ZonMw, all paid to the institution.

Prof. Schuurman is a consultant for Medtronic and Boston Scientific on educational matters.

The other authors report no financial disclosures.
