# Supplementary material for: Thalamic local field potentials recorded using the deep brain stimulation pulse generator
Source: Clin Neurophysiol Pract. 2022 Mar 10;7:103–6. doi: 10.1016/j.cnp.2022.03.002 (PMC8956842; doi:10.1016/j.cnp.2022.03.002)
Supplement: Supplementary data 1 [file mmc1.docx]

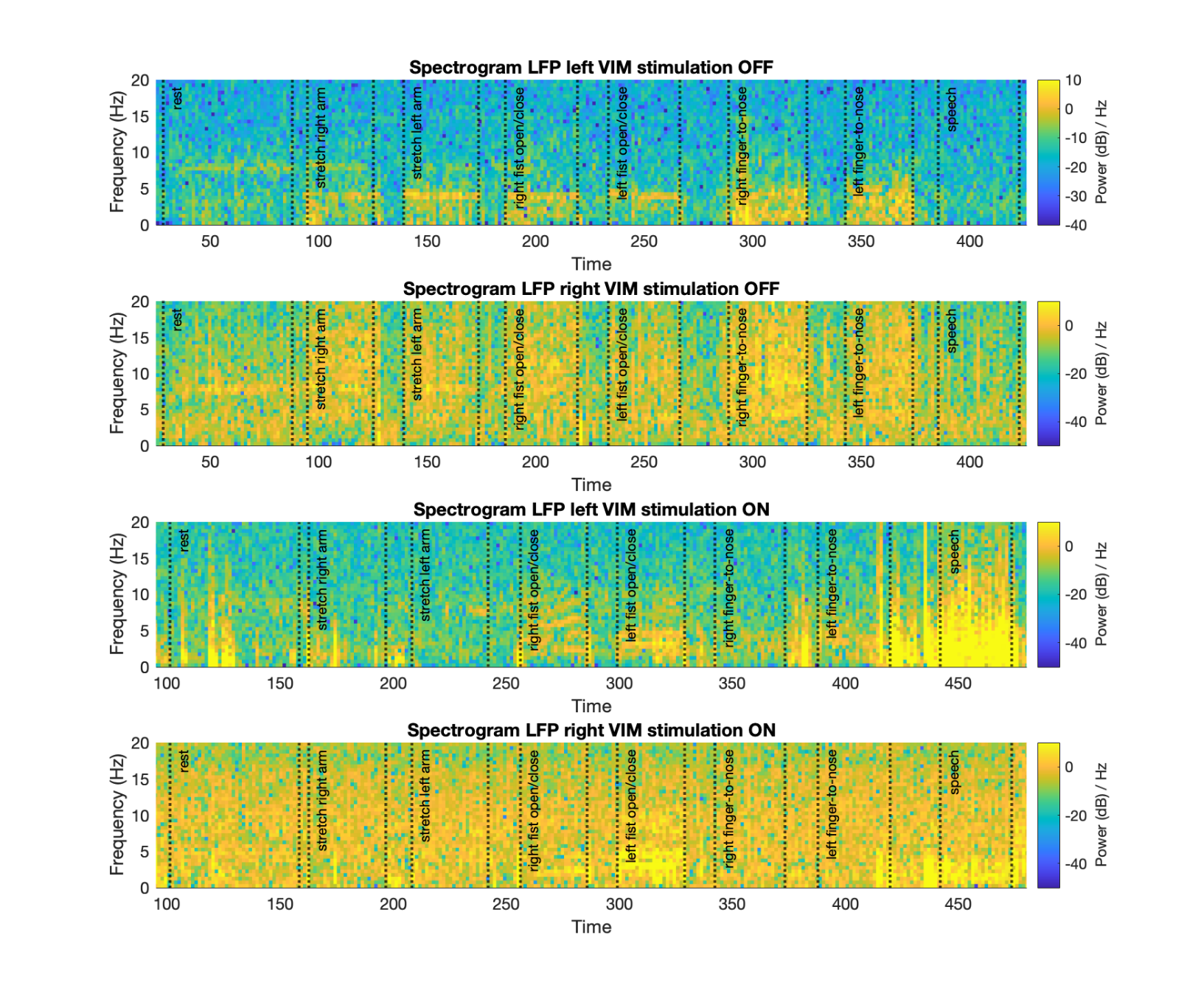


**Supplementary figure 1.** A. Spectrogram (0-20 Hz) of the LFP of the *left* Vim nucleus with stimulation at 0.0 mA (OFF). B. Spectrogram (0-20 Hz) spectrogram of the LFP of the *right* Vim nucleus with stimulation at 0.0 mA (OFF). C. Spectrogram (0-20 Hz) spectrogram of the LFP of the left Vim nucleus with stimulation at 1.4 mA (ON). D. Spectrogram (0-20 Hz) spectrogram of the LFP of the right Vim nucleus with stimulation at 1.2 mA (ON).
